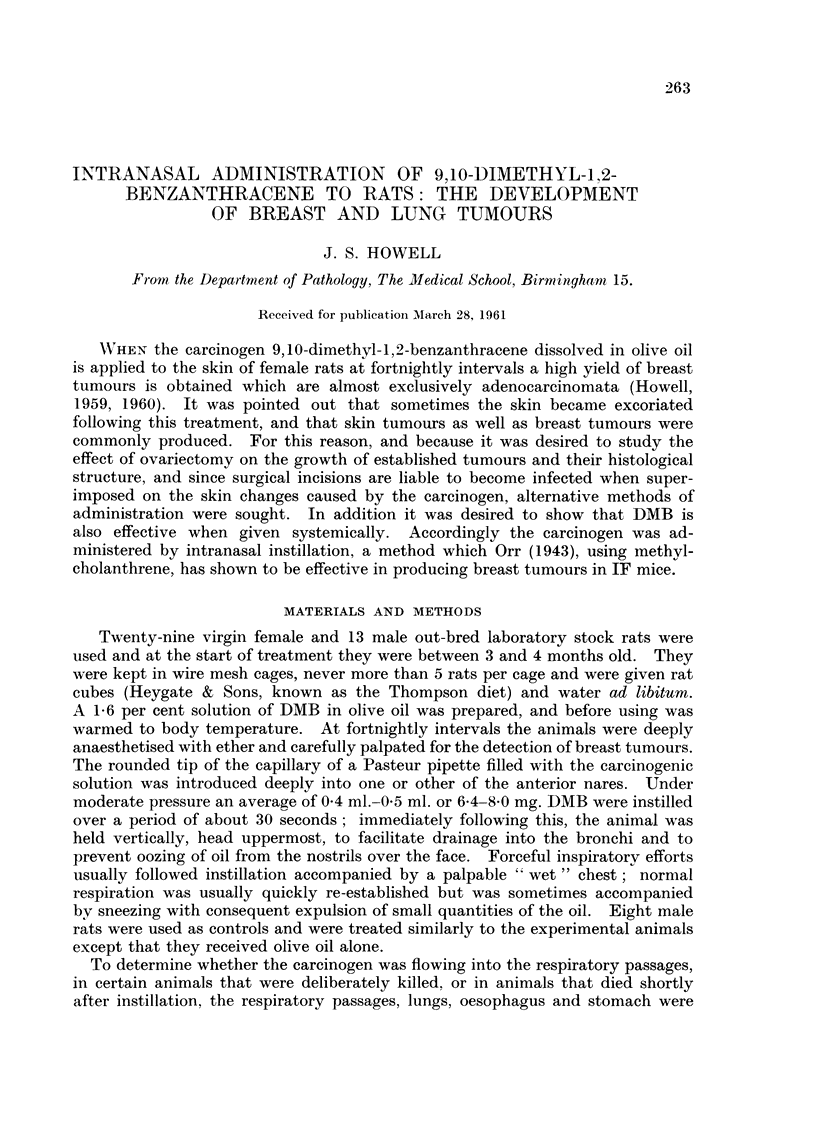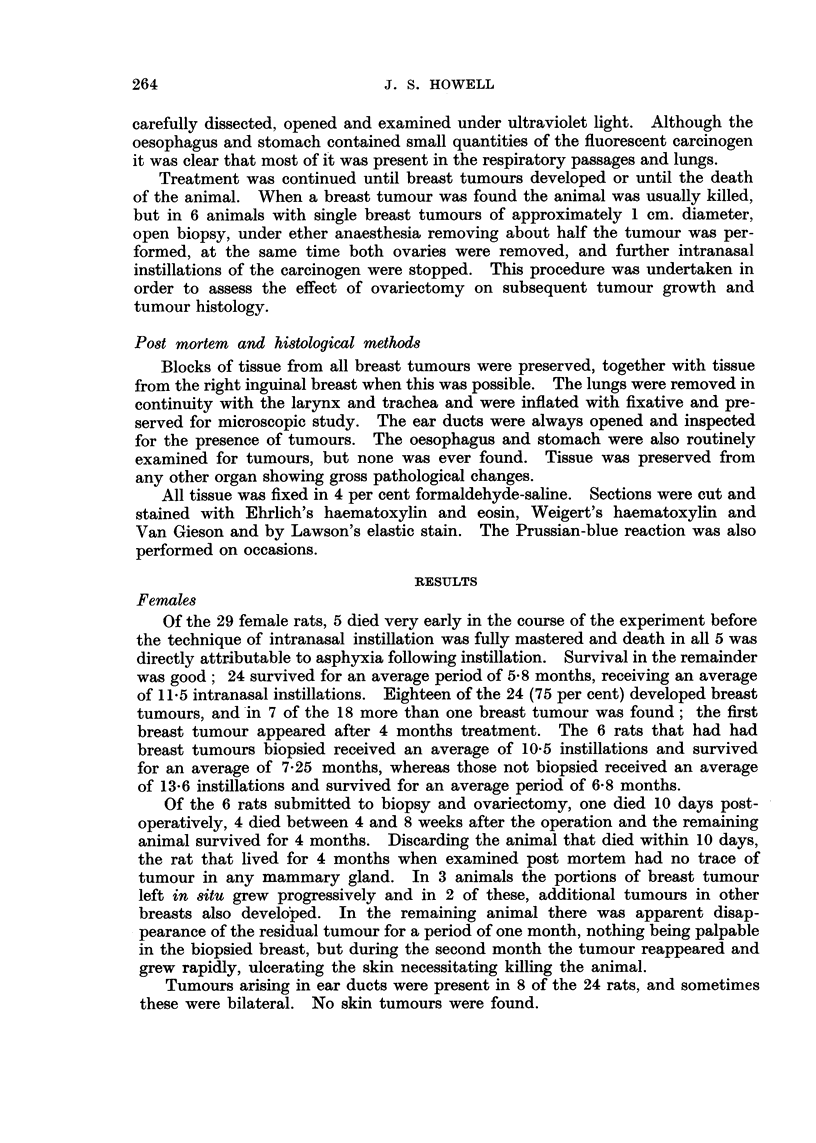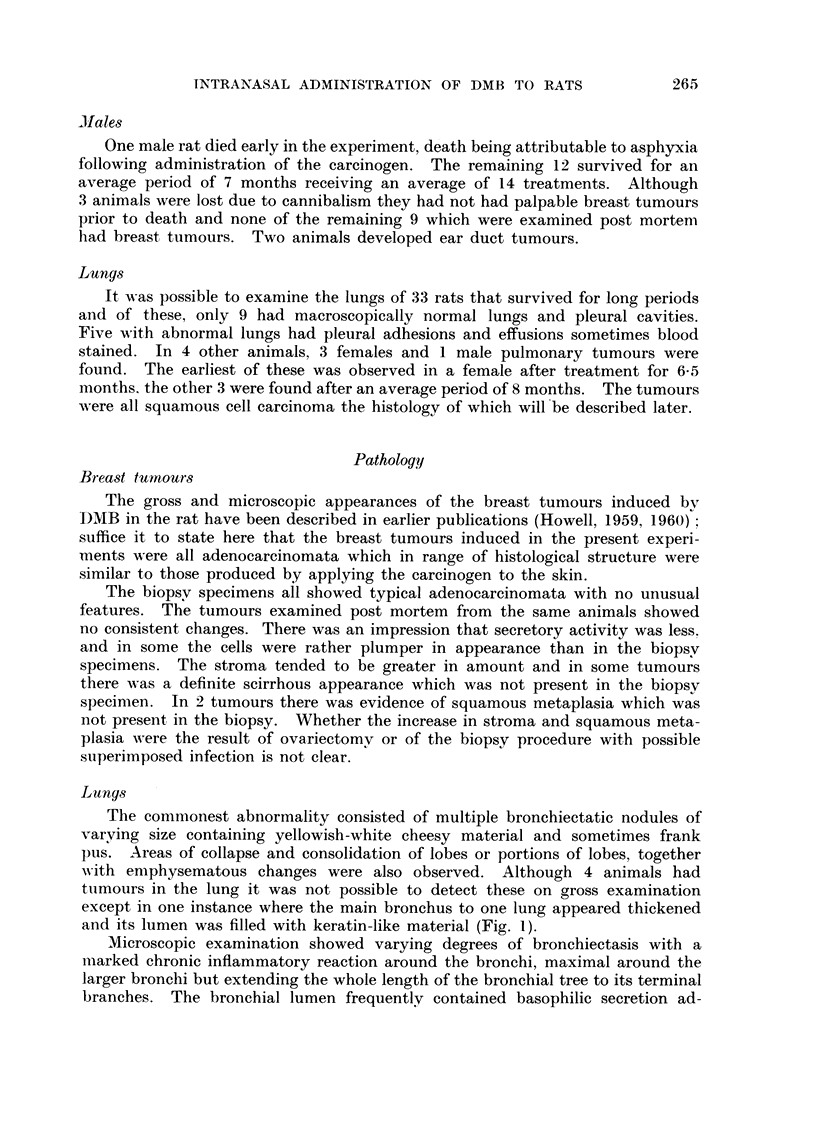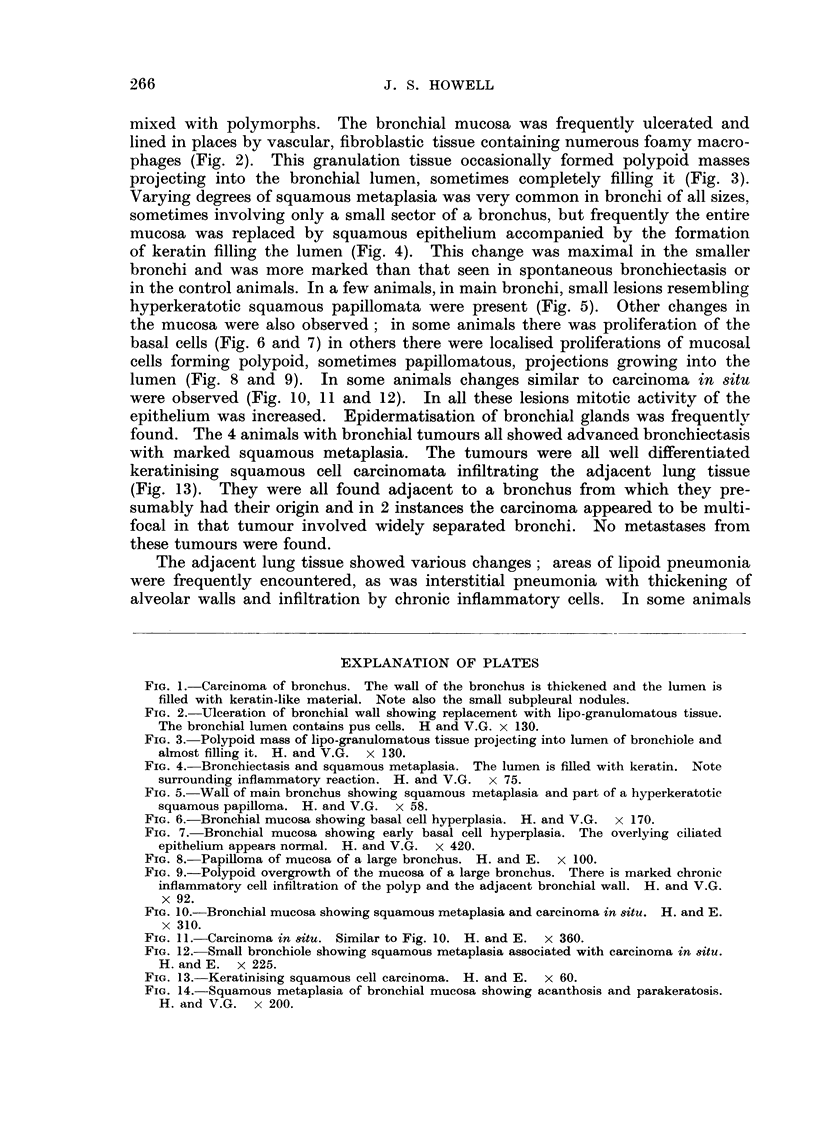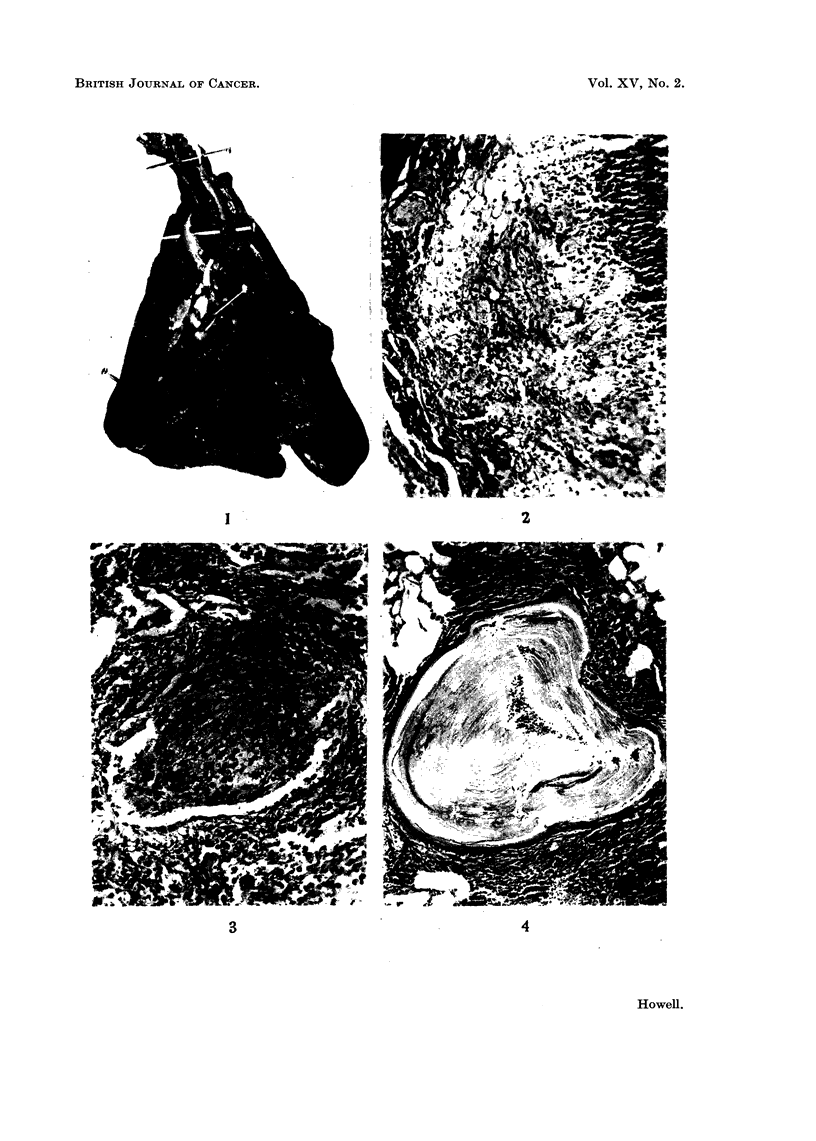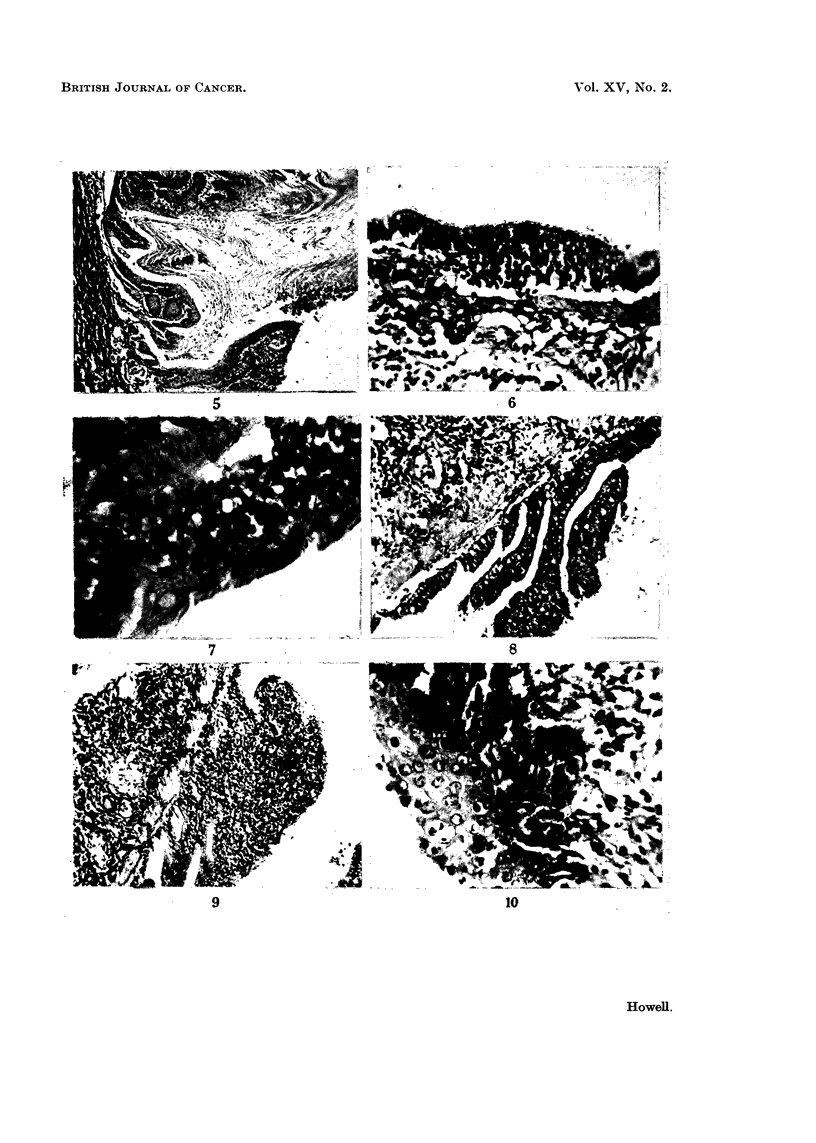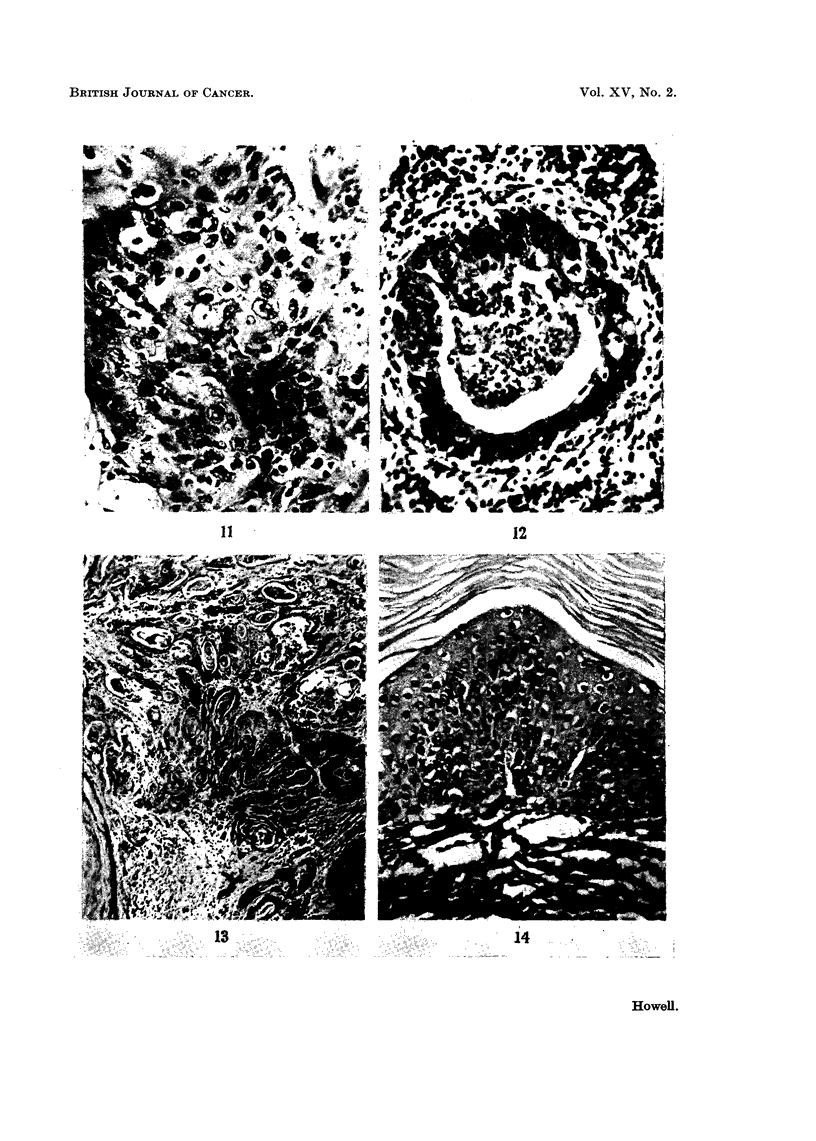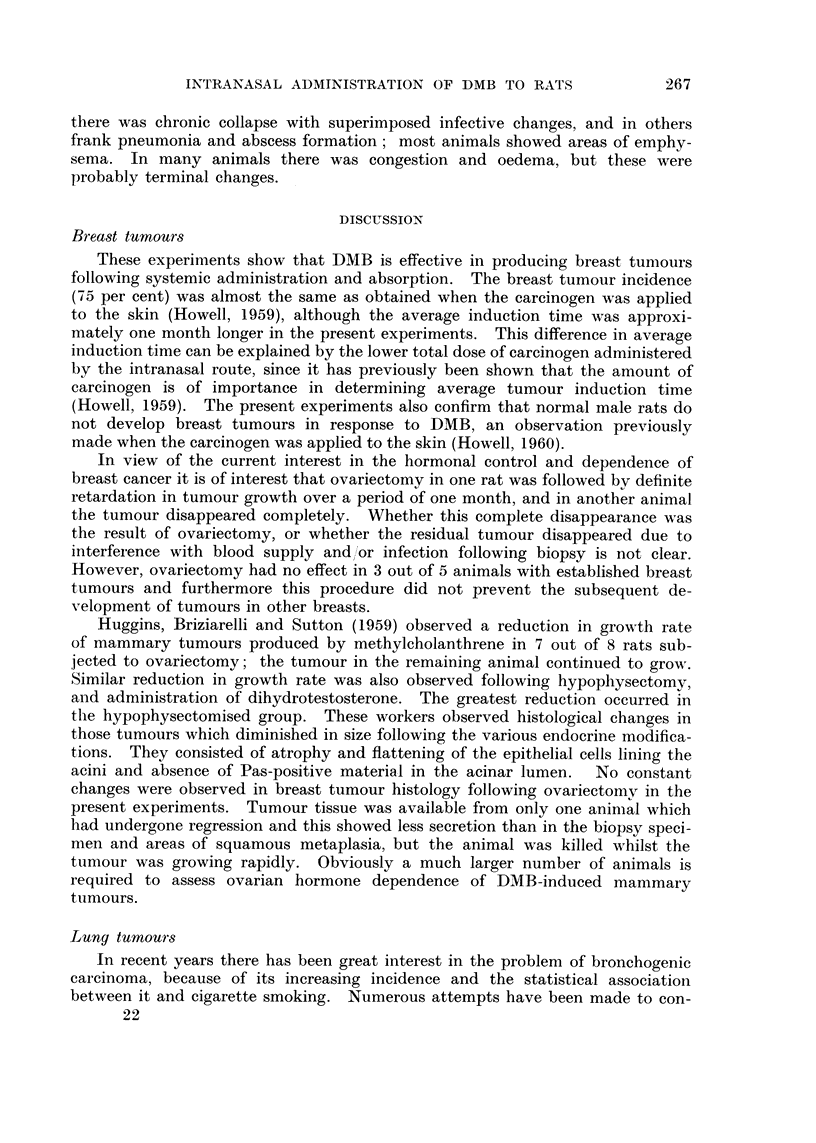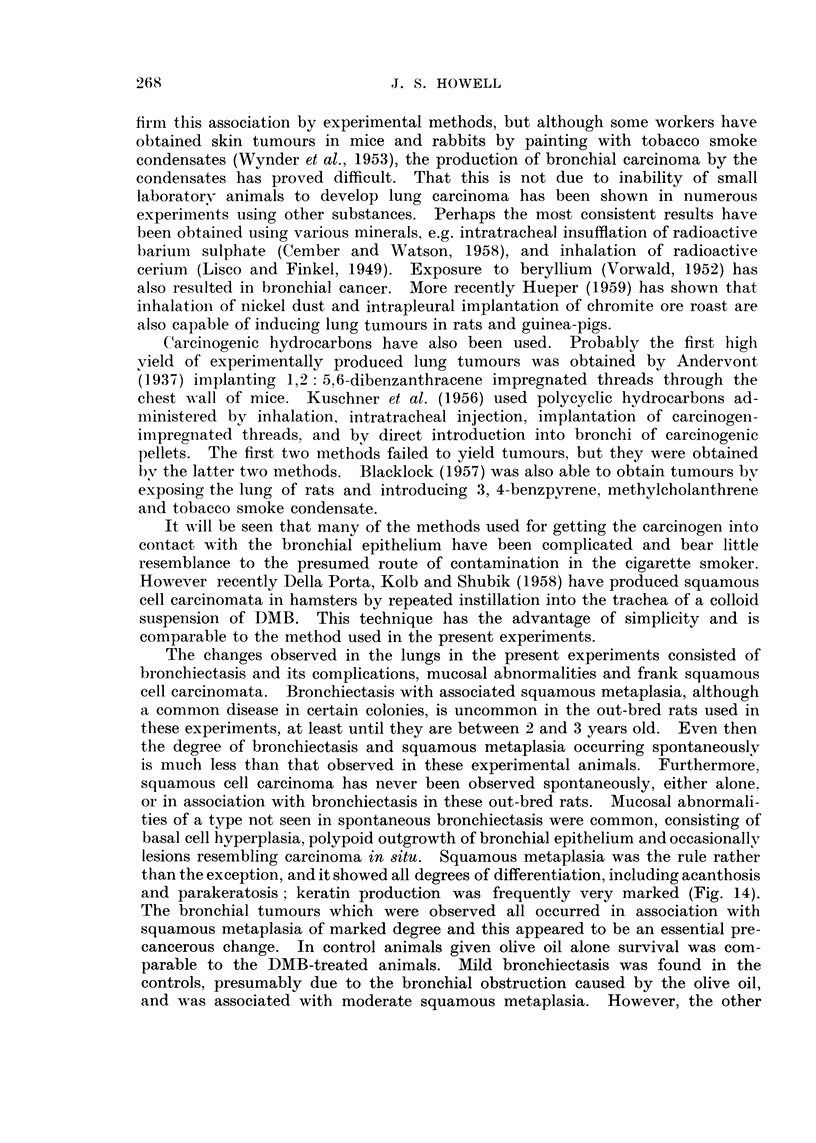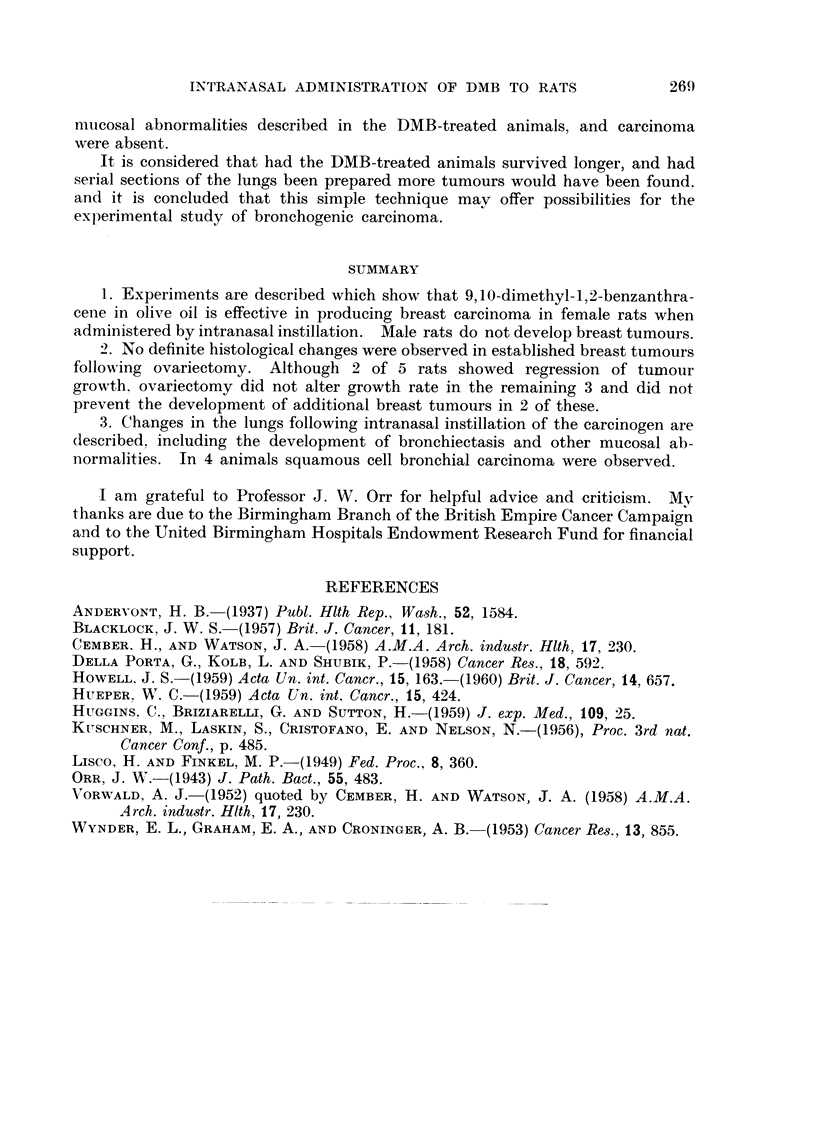# Intranasal Administration of 9,10-dimethyl-1,2-Benzanthracene to Rats: the Development of Breast and Lung Tumours

**DOI:** 10.1038/bjc.1961.33

**Published:** 1961-06

**Authors:** J. S. Howell

## Abstract

**Images:**


### INTRANASAL ADMINISTRATION OF 9,10-DIMETHYL-1,2-BENZANTHRACENE TO RATS: THE DEVELOPMENT OF BREAST AND LUNG TUMOURS

J. S. HOWELL

*From the Department of Pathology, The Medical School, Birmingham* 15.

Received for publication March 28, 1961

WHEN the carcinogen 9,10-dimethyl-1,2-benzanthracene dissolved in olive oil is applied to the skin of female rats at fortnightly intervals a high yield of breast tumours is obtained which are almost exclusively adenocarcinomata (Howell, 1959, 1960). It was pointed out that sometimes the skin became excoriated following this treatment, and that skin tumours as well as breast tumours were commonly produced. For this reason, and because it was desired to study the effect of ovariectomy on the growth of established tumours and their histological structure, and since surgical incisions are liable to become infected when superimposed on the skin changes caused by the carcinogen, alternative methods of administration were sought. In addition it was desired to show that DMB is also effective when given systemically. Accordingly the carcinogen was administered by intranasal instillation, a method which Orr (1943), using methylcholanthrene, has shown to be effective in producing breast tumours in IF mice.

### MATERIALS AND METHODS

Twenty-nine virgin female and 13 male out-bred laboratory stock rats were used and at the start of treatment they were between 3 and 4 months old. They were kept in wire mesh cages, never more than 5 rats per cage and were given rat cubes (Heygate & Sons, known as the Thompson diet) and water *ad libitum.* A 1·6 per cent solution of DMB in olive oil was prepared, and before using was warmed to body temperature. At fortnightly intervals the animals were deeply anaesthetised with ether and carefully palpated for the detection of breast tumours. The rounded tip of the capillary of a Pasteur pipette filled with the carcinogenic solution was introduced deeply into one or other of the anterior nares. Under moderate pressure an average of 0·4 ml.–0·5 ml. or 6·4–8·0 mg. DMB were instilled over a period of about 30 seconds ; immediately following this, the animal was held vertically, head uppermost, to facilitate drainage into the bronchi and to prevent oozing of oil from the nostrils over the face. Forceful inspiratory efforts usually followed instillation accompanied by a palpable " wet " chest ; normal respiration was usually quickly re-established but was sometimes accompanied by sneezing with consequent expulsion of small quantities of the oil. Eight male rats were used as controls and were treated similarly to the experimental animals except that they received olive oil alone.

To determine whether the carcinogen was flowing into the respiratory passages, in certain animals that were deliberately killed, or in animals that died shortly after instillation, the respiratory passages, lungs, oesophagus and stomach were carefully dissected, opened and examined under ultraviolet light. Although the oesophagus and stomach contained small quantities of the fluorescent carcinogen it was clear that most of it was present in the respiratory passages and lungs.

Treatment was continued until breast tumours developed or until the death of the animal. When a breast tumour was found the animal was usually killed, but in 6 animals with single breast tumours of approximately 1 cm. diameter, open biopsy, under ether anaesthesia removing about half the tumour was performed, at the same time both ovaries were removed, and further intranasal instillations of the carcinogen were stopped. This procedure was undertaken in order to assess the effect of ovariectomy on subsequent tumour growth and tumour histology.

*Post mortem and histological methods*

Blocks of tissue from all breast tumours were preserved, together with tissue from the right inguinal breast when this was possible. The lungs were removed in continuity with the larynx and trachea and were inflated with fixative and preserved for microscopic study. The ear ducts were always opened and inspected for the presence of tumours. The oesophagus and stomach were also routinely examined for tumours, but none was ever found. Tissue was preserved from any other organ showing gross pathological changes.

All tissue was fixed in 4 per cent formaldehyde-saline. Sections were cut and stained with Ehrlich's haematoxylin and eosin, Weigert's haematoxylin and Van Gieson and by Lawson's elastic stain. The Prussian-blue reaction was also performed on occasions.

### RESULTS

*Females*

Of the 29 female rats, 5 died very early in the course of the experiment before the technique of intranasal instillation was fully mastered and death in all 5 was directly attributable to asphyxia following instillation. Survival in the remainder was good ; 24 survived for an average period of 5·8 months, receiving an average of 11·5 intranasal instillations. Eighteen of the 24 (75 per cent) developed breast tumours, and in 7 of the 18 more than one breast tumour was found ; the first breast tumour appeared after 4 months treatment. The 6 rats that had had breast tumours biopsied received an average of 10·5 instillations and survived for an average of 7·25 months, whereas those not biopsied received an average of 13·6 instillations and survived for an average period of 6·8 months.

Of the 6 rats submitted to biopsy and ovariectomy, one died 10 days post-operatively, 4 died between 4 and 8 weeks after the operation and the remaining animal survived for 4 months. Discarding the animal that died within 10 days, the rat that lived for 4 months when examined post mortem had no trace of tumour in any mammary gland. In 3 animals the portions of breast tumour left *in situ* grew progressively and in 2 of these, additional tumours in other breasts also developed. In the remaining animal there was apparent disappearance of the residual tumour for a period of one month, nothing being palpable in the biopsied breast, but during the second month the tumour reappeared and grew rapidly, ulcerating the skin necessitating killing the animal.

Tumours arising in ear ducts were present in 8 of the 24 rats, and sometimes these were bilateral. No skin tumours were found.

*Males*

One male rat died early in the experiment, death being attributable to asphyxia following administration of the carcinogen. The remaining 12 survived for an average period of 7 months receiving an average of 14 treatments. Although 3 animals were lost due to cannibalism they had not had palpable breast tumours prior to death and none of the remaining 9 which were examined post mortem had breast tumours. Two animals developed ear duct tumours.

*Lungs*

It was possible to examine the lungs of 33 rats that survived for long periods and of these, only 9 had macroscopically normal lungs and pleural cavities. Five with abnormal lungs had pleural adhesions and effusions sometimes blood stained. In 4 other animals, 3 females and 1 male pulmonary tumours were found. The earliest of these was observed in a female after treatment for 6·5 months, the other 3 were found after an average period of 8 months. The tumours were all squamous cell carcinoma the histology of which will be described later.

### *Pathology*

*Breast tumours*

The gross and microscopic appearances of the breast tumours induced by DMB in the rat have been described in earlier publications (Howell, 1959, 1960); suffice it to state here that the breast tumours induced in the present experiments were all adenocarcinomata which in range of histological structure were similar to those produced by applying the carcinogen to the skin.

The biopsy specimens all showed typical adenocarcinomata with no unusual features. The tumours examined post mortem from the same animals showed no consistent changes. There was an impression that secretory activity was less, and in some the cells were rather plumper in appearance than in the biopsy specimens. The stroma tended to be greater in amount and in some tumours there was a definite scirrhous appearance which was not present in the biopsy specimen. In 2 tumours there was evidence of squamous metaplasia which was not present in the biopsy. Whether the increase in stroma and squamous metaplasia were the result of ovariectomy or of the biopsy procedure with possible superimposed infection is not clear.

*Lungs*

The commonest abnormality consisted of multiple bronchiectatic nodules of varying size containing yellowish-white cheesy material and sometimes frank pus. Areas of collapse and consolidation of lobes or portions of lobes, together with emphysematous changes were also observed. Although 4 animals had tumours in the lung it was not possible to detect these on gross examination except in one instance where the main bronchus to one lung appeared thickened and its lumen was filled with keratin-like material (Fig. 1).

Microscopic examination showed varying degrees of bronchiectasis with a marked chronic inflammatory reaction around the bronchi, maximal around the larger bronchi but extending the whole length of the bronchial tree to its terminal branches. The bronchial lumen frequently contained basophilic secretion admixed with polymorphs. The bronchial mucosa was frequently ulcerated and lined in places by vascular, fibroblastic tissue containing numerous foamy macrophages (Fig. 2). This granulation tissue occasionally formed polypoid masses projecting into the bronchial lumen, sometimes completely filling it (Fig. 3). Varying degrees of squamous metaplasia was very common in bronchi of all sizes, sometimes involving only a small sector of a bronchus, but frequently the entire mucosa was replaced by squamous epithelium accompanied by the formation of keratin filling the lumen (Fig. 4). This change was maximal in the smaller bronchi and was more marked than that seen in spontaneous bronchiectasis or in the control animals. In a few animals, in main bronchi, small lesions resembling hyperkeratotic squamous papillomata were present (Fig. 5). Other changes in the mucosa were also observed ; in some animals there was proliferation of the basal cells (Fig. 6 and 7) in others there were localised proliferations of mucosal cells forming polypoid, sometimes papillomatous, projections growing into the lumen (Fig. 8 and 9). In some animals changes similar to carcinoma *in situ* were observed (Fig. 10, 11 and 12). In all these lesions mitotic activity of the epithelium was increased. Epidermatisation of bronchial glands was frequently found. The 4 animals with bronchial tumours all showed advanced bronchiectasis with marked squamous metaplasia. The tumours were all well differentiated keratinising squamous cell carcinomata infiltrating the adjacent lung tissue (Fig. 13). They were all found adjacent to a bronchus from which they presumably had their origin and in 2 instances the carcinoma appeared to be multifocal in that tumour involved widely separated bronchi. No metastases from these tumours were found.

The adjacent lung tissue showed various changes ; areas of lipoid pneumonia were frequently encountered, as was interstitial pneumonia with thickening of alveolar walls and infiltration by chronic inflammatory cells. In some animals

---

EXPLANATION OF PLATES

FIG. 1.—Carcinoma of bronchus. The wall of the bronchus is thickened and the lumen is filled with keratin-like material. Note also the small subpleural nodules.

FIG. 2.—Ulceration of bronchial wall showing replacement with lipo-granulomatous tissue. The bronchial lumen contains pus cells. H and V.G. × 130.

FIG. 3.—Polypoid mass of lipo-granulomatous tissue projecting into lumen of bronchiole and almost filling it. H. and V.G. × 130.

FIG. 4.—Bronchiectasis and squamous metaplasia. The lumen is filled with keratin. Note surrounding inflammatory reaction. H. and V.G. × 75.

FIG. 5.—Wall of main bronchus showing squamous metaplasia and part of a hyperkeratotic squamous papilloma. H. and V.G. × 58.

FIG. 6.—Bronchial mucosa showing basal cell hyperplasia. H. and V.G. × 170.

FIG. 7.—Bronchial mucosa showing early basal cell hyperplasia. The overlying ciliated epithelium appears normal. H. and V.G. × 420.

FIG. 8.—Papilloma of mucosa of a large bronchus. H. and E. × 100.

FIG. 9.—Polypoid overgrowth of the mucosa of a large bronchus. There is marked chronic inflammatory cell infiltration of the polyp and the adjacent bronchial wall. H. and V.G. × 92.

FIG. 10.—Bronchial mucosa showing squamous metaplasia and carcinoma *in situ*. H. and E. × 310.

FIG. 11.—Carcinoma *in situ*. Similar to Fig. 10. H. and E. × 360.

FIG. 12.—Small bronchiole showing squamous metaplasia associated with carcinoma *in situ*. H. and E. × 225.

FIG. 13.—Keratinising squamous cell carcinoma. H. and E. × 60.

FIG. 14.—Squamous metaplasia of bronchial mucosa showing acanthosis and parakeratosis. H. and V.G. × 200.

1

2

3

4

Howell.

5

6

7

8

9

10

Howell.

11

12

13

14

Howell.

there was chronic collapse with superimposed infective changes, and in others frank pneumonia and abscess formation ; most animals showed areas of emphysema. In many animals there was congestion and oedema, but these were probably terminal changes.

### DISCUSSION

*Breast tumours*

These experiments show that DMB is effective in producing breast tumours following systemic administration and absorption. The breast tumour incidence (75 per cent) was almost the same as obtained when the carcinogen was applied to the skin (Howell, 1959), although the average induction time was approximately one month longer in the present experiments. This difference in average induction time can be explained by the lower total dose of carcinogen administered by the intranasal route, since it has previously been shown that the amount of carcinogen is of importance in determining average tumour induction time (Howell, 1959). The present experiments also confirm that normal male rats do not develop breast tumours in response to DMB, an observation previously made when the carcinogen was applied to the skin (Howell, 1960).

In view of the current interest in the hormonal control and dependence of breast cancer it is of interest that ovariectomy in one rat was followed by definite retardation in tumour growth over a period of one month, and in another animal the tumour disappeared completely. Whether this complete disappearance was the result of ovariectomy, or whether the residual tumour disappeared due to interference with blood supply and/or infection following biopsy is not clear. However, ovariectomy had no effect in 3 out of 5 animals with established breast tumours and furthermore this procedure did not prevent the subsequent development of tumours in other breasts.

Huggins, Briziarelli and Sutton (1959) observed a reduction in growth rate of mammary tumours produced by methylcholanthrene in 7 out of 8 rats subjected to ovariectomy; the tumour in the remaining animal continued to grow. Similar reduction in growth rate was also observed following hypophysectomy, and administration of dihydrotestosterone. The greatest reduction occurred in the hypophysectomised group. These workers observed histological changes in those tumours which diminished in size following the various endocrine modifications. They consisted of atrophy and flattening of the epithelial cells lining the acini and absence of Pas-positive material in the acinar lumen. No constant changes were observed in breast tumour histology following ovariectomy in the present experiments. Tumour tissue was available from only one animal which had undergone regression and this showed less secretion than in the biopsy specimen and areas of squamous metaplasia, but the animal was killed whilst the tumour was growing rapidly. Obviously a much larger number of animals is required to assess ovarian hormone dependence of DMB-induced mammary tumours.

*Lung tumours*

In recent years there has been great interest in the problem of bronchogenic carcinoma, because of its increasing incidence and the statistical association between it and cigarette smoking. Numerous attempts have been made to confirm this association by experimental methods, but although some workers have obtained skin tumours in mice and rabbits by painting with tobacco smoke condensates (Wynder *et al.*, 1953), the production of bronchial carcinoma by the condensates has proved difficult. That this is not due to inability of small laboratory animals to develop lung carcinoma has been shown in numerous experiments using other substances. Perhaps the most consistent results have been obtained using various minerals, e.g. intratracheal insufflation of radioactive barium sulphate (Cember and Watson, 1958), and inhalation of radioactive cerium (Lisco and Finkel, 1949). Exposure to beryllium (Vorwald, 1952) has also resulted in bronchial cancer. More recently Hueper (1959) has shown that inhalation of nickel dust and intrapleural implantation of chromite ore roast are also capable of inducing lung tumours in rats and guinea-pigs.

Carcinogenic hydrocarbons have also been used. Probably the first high yield of experimentally produced lung tumours was obtained by Andervont (1937) implanting 1,2 : 5,6-dibenzanthracene impregnated threads through the chest wall of mice. Kuschner *et al.* (1956) used polycyclic hydrocarbons administered by inhalation, intratracheal injection, implantation of carcinogen-impregnated threads, and by direct introduction into bronchi of carcinogenic pellets. The first two methods failed to yield tumours, but they were obtained by the latter two methods. Blacklock (1957) was also able to obtain tumours by exposing the lung of rats and introducing 3, 4-benzpyrene, methylcholanthrene and tobacco smoke condensate.

It will be seen that many of the methods used for getting the carcinogen into contact with the bronchial epithelium have been complicated and bear little resemblance to the presumed route of contamination in the cigarette smoker. However recently Della Porta, Kolb and Shubik (1958) have produced squamous cell carcinomata in hamsters by repeated instillation into the trachea of a colloid suspension of DMB. This technique has the advantage of simplicity and is comparable to the method used in the present experiments.

The changes observed in the lungs in the present experiments consisted of bronchiectasis and its complications, mucosal abnormalities and frank squamous cell carcinomata. Bronchiectasis with associated squamous metaplasia, although a common disease in certain colonies, is uncommon in the out-bred rats used in these experiments, at least until they are between 2 and 3 years old. Even then the degree of bronchiectasis and squamous metaplasia occurring spontaneously is much less than that observed in these experimental animals. Furthermore, squamous cell carcinoma has never been observed spontaneously, either alone, or in association with bronchiectasis in these out-bred rats. Mucosal abnormalities of a type not seen in spontaneous bronchiectasis were common, consisting of basal cell hyperplasia, polypoid outgrowth of bronchial epithelium and occasionally lesions resembling carcinoma *in situ*. Squamous metaplasia was the rule rather than the exception, and it showed all degrees of differentiation, including acanthosis and parakeratosis ; keratin production was frequently very marked (Fig. 14). The bronchial tumours which were observed all occurred in association with squamous metaplasia of marked degree and this appeared to be an essential precancerous change. In control animals given olive oil alone survival was comparable to the DMB-treated animals. Mild bronchiectasis was found in the controls, presumably due to the bronchial obstruction caused by the olive oil, and was associated with moderate squamous metaplasia. However, the other mucosal abnormalities described in the DMB-treated animals, and carcinoma were absent.

It is considered that had the DMB-treated animals survived longer, and had serial sections of the lungs been prepared more tumours would have been found, and it is concluded that this simple technique may offer possibilities for the experimental study of bronchogenic carcinoma.

### SUMMARY

1. Experiments are described which show that 9,10-dimethyl-1,2-benzanthracene in olive oil is effective in producing breast carcinoma in female rats when administered by intranasal instillation. Male rats do not develop breast tumours.

2. No definite histological changes were observed in established breast tumours following ovariectomy. Although 2 of 5 rats showed regression of tumour growth, ovariectomy did not alter growth rate in the remaining 3 and did not prevent the development of additional breast tumours in 2 of these.

3. Changes in the lungs following intranasal instillation of the carcinogen are described, including the development of bronchiectasis and other mucosal abnormalities. In 4 animals squamous cell bronchial carcinoma were observed.

I am grateful to Professor J. W. Orr for helpful advice and criticism. My thanks are due to the Birmingham Branch of the British Empire Cancer Campaign and to the United Birmingham Hospitals Endowment Research Fund for financial support.

### REFERENCES

ANDERVONT, H. B.—(1937) *Publ. Hlth Rep., Wash.*, **52**, 1584.
BLACKLOCK, J. W. S.—(1957) *Brit. J. Cancer*, **11**, 181.
CEMBER, H., AND WATSON, J. A.—(1958) *A.M.A. Arch. industr. Hlth*, **17**, 230.
DELLA PORTA, G., KOLB, L. AND SHUBIK, P.—(1958) *Cancer Res.*, **18**, 592.
HOWELL, J. S.—(1959) *Acta Un. int. Cancr.*, **15**, 163.—(1960) *Brit. J. Cancer*, **14**, 657.
HUEPER, W. C.—(1959) *Acta Un. int. Cancr.*, **15**, 424.
HUGGINS, C., BRIZIARELLI, G. AND SUTTON, H.—(1959) *J. exp. Med.*, **109**, 25.
KUSCHNER, M., LASKIN, S., CRISTOFANO, E. AND NELSON, N.—(1956), *Proc. 3rd nat. Cancer Conf.*, p. 485.
LISCO, H. AND FINKEL, M. P.—(1949) *Fed. Proc.*, **8**, 360.
ORR, J. W.—(1943) *J. Path. Bact.*, **55**, 483.
VORWALD, A. J.—(1952) quoted by CEMBER, H. AND WATSON, J. A. (1958) *A.M.A. Arch. industr. Hlth*, **17**, 230.
WYNDER, E. L., GRAHAM, E. A., AND CRONINGER, A. B.—(1953) *Cancer Res.*, **13**, 855.